# Rapid detection of SMARCB1 sequence variation using high resolution melting

**DOI:** 10.1186/1471-2407-9-437

**Published:** 2009-12-13

**Authors:** Vinod Dagar, Chung-Wo Chow, David M Ashley, Elizabeth M Algar

**Affiliations:** 1 Molecular Oncology Laboratory, Children's Cancer Centre, Murdoch Children's Research Institute, Royal Children's Hospital, Flemington Rd, Parkville, 3052, Australia; 2 Anatomical Pathology, Royal Children's Hospital, Flemington Rd, Parkville, 3052, Australia; 3 Department of Paediatrics, University of Melbourne, Parkville, 3052, Australia

## Abstract

**Background:**

Rhabdoid tumors are rare cancers of early childhood arising in the kidney, central nervous system and other organs. The majority are caused by somatic inactivating mutations or deletions affecting the tumor suppressor locus SMARCB1 [OMIM 601607]. Germ-line SMARCB1 inactivation has been reported in association with rhabdoid tumor, epitheloid sarcoma and familial schwannomatosis, underscoring the importance of accurate mutation screening to ascertain recurrence and transmission risks. We describe a rapid and sensitive diagnostic screening method, using high resolution melting (HRM), for detecting sequence variations in SMARCB1.

**Methods:**

Amplicons, encompassing the nine coding exons of SMARCB1, flanking splice site sequences and the 5' and 3' UTR, were screened by both HRM and direct DNA sequencing to establish the reliability of HRM as a primary mutation screening tool. Reaction conditions were optimized with commercially available HRM mixes.

**Results:**

The false negative rate for detecting sequence variants by HRM in our sample series was zero. Nine amplicons out of a total of 140 (6.4%) showed variant melt profiles that were subsequently shown to be false positive. Overall nine distinct pathogenic SMARCB1 mutations were identified in a total of 19 possible rhabdoid tumors. Two tumors had two distinct mutations and two harbored SMARCB1 deletion. Other mutations were nonsense or frame-shifts. The detection sensitivity of the HRM screening method was influenced by both sequence context and specific nucleotide change and varied from 1: 4 to 1:1000 (variant to wild-type DNA). A novel method involving digital HRM, followed by re-sequencing, was used to confirm mutations in tumor specimens containing associated normal tissue.

**Conclusions:**

This is the first report describing SMARCB1 mutation screening using HRM. HRM is a rapid, sensitive and inexpensive screening technology that is likely to be widely adopted in diagnostic laboratories to facilitate whole gene mutation screening.

## Background

Rhabdoid tumors (RT) are a rare tumour of infancy and early childhood and mainly arise in the kidney and in the central nervous system where they are referred to as atypical teratoid rhabdoid tumors (AT/RT). The majority of RT (85%) are characterized by deletion of chromosome 22q, and associated inactivating mutations or deletions, affecting tumour suppressor *SMARCB1 *[OMIM 601607] [1,2]. Few other consistent cytogenetic abnormalities have been described in RT. Rhabdoid tumors are highly resistant to conventional chemotherapies and to radiotherapy and patients frequently succumb rapidly to the disease.

Most histopathology laboratories confirm the diagnosis of rhabdoid tumor based on loss of immunostaining for SMARCB1 protein in the tumor cell nuclei and this correlates well with the presence of inactivating SMARCB1 mutations and with homozygous deletion of the gene [3,4]. However there have been several reports of germ-line mutations in SMARCB1 in association with rhabdoid tumor and with other tumors including epitheloid sarcoma and familial schwannomatosis [4-9]. Germ-line mutations have been described in patients where the rhabdoid tumor has arisen at multiple sites ("rhabdoid predisposition syndrome") and in cases of familial rhabdoid tumor where more than one child in a family is affected [10]. These reports underscore the importance of somatic tumor SMARCB1 mutation screening in these cases so that follow-up germline studies may be performed to ascertain both recurrence risk in affected cases and the transmission risk within affected families.

In this report we describe the validation of a rapid and sensitive diagnostic screening method, using high resolution melting (HRM), for detecting sequence variations in SMARCB1. HRM relies on the principle that DNA sequence variation produces altered melting characteristics in DNA amplicons. Melting data is captured as a fluorescent signal from intercalating dyes that are evenly distributed within double-stranded DNA during PCR amplification, and then later released from the amplicon during the melting phase [11]. The PCR and melting steps occur consecutively in a closed tube. Amplicons with melting profiles that are shifted relative to reference amplicons of known sequence are selected and any sequence variation is confirmed by direct amplicon sequencing. The SMARCB1 mutation screening method we describe is rapid, sensitive and inexpensive and will be useful for both diagnostic molecular pathology and molecular genetics laboratories.

## Methods

Rhabdoid tumors were submitted to the laboratory for somatic SMARCB1 mutation screening. The validation study described was granted institutional approval in accordance with the policies of the Human Ethics Research Committee of the Royal Children's Hospital, approval number CA29031. DNA was extracted from fresh frozen tissue or from formalin fixed paraffin-embedded (FFPE) tissue sections using the DNeasy kit (Qiagen) according to the manufacturer's instructions.

Primers for amplification of genomic DNA were as described in [1], with modifications to the exon 1 and exon 5 primer pairs. Exon 1 was divided into two overlapping fragments, Exon 1-1 and Exon 1-2. Primer sequences, amplicon sizes and annealing temperatures are shown in Table 1.

**Table 1:** PCR conditions for SMARCB1 mutation screening

Exon	Primer	Amplicon size bp	Annealing temperature
**5' Exon 1-1**	INI1F actgagggcggcctggtcgt Snf1Rb ccgaaggtcttgctcagcgc	222	65°C

**3' Exon 1-2**	Snf1Fb ctctgccgccgcaatgatgatg INI1R cgacacgcccactaggccac	261	64°C

**Exon 2**	INI12F ctgcgacccttataatgagc INI12R gcgagtggttttgaaacagg	213	58°C

**Exon 3**	INI13F accagcagagtgacccagtg INI13R agagatgccctggccaggaa	195	60°C

**Exon 4**	INI14F tcgagcctgacagaggtacagtg INI14R gaatcagcacggagggtgagt	284	60°C

**Exon 5**	INI15F ttgcatacctagggctccgg INI15R cacgtaacacacaggggttg	238	60°C

**Exon 6**	INI16F tggtgcaatctcttggcatc INI16R tcagtgctccatgatgacac	277	60°C

**Exon 7**	INI17F tgggctgcaaaagctctaac INI17R cgctcacacagagaagtctt	312	60°C

**Exon 8**	INI18F atccactgggtgccagcagt INI18R tctgcctggaaagccaggtg	313	60°C

**Exon 9**	INI19F ccctgtagagccttgggaag INI19R gcctctgtccttgccagaag	200	60°C

PCR and HRM were performed on a Rotorgene 6000 (Qiagen, Hilden Germany). Cycling was for 45 cycles with denaturation for 15 seconds at 95°C, annealing and extension for 10 seconds each. The extension temperature was 72°C and the annealing temperature varied according to the amplicon. PCR mixes used for method validation were: 1 × Sensimix (Quantace), 10 picomole of each primer and 0.8 uL EvaGreen Dye (supplied with Sensimix) in a 20 ul total reaction volume. 30 ng of DNA was added to each reaction. Reactions were set up in duplicate. For exon 1, HRM was performed with ramping from 82°C to 97°C rising by 0.2°C at each step, for exons 2, 3, 4, 5, 6, 7 and 8, HRM was performed with ramping from 75°C to 90°C rising by 0.2°C at each step, and for exon 9 ramping was from 80°C to 95°C rising by 0.2°C at each step. The confidence percentage threshold of the Rotorgene 6000 software was set at 90% for variant scoring. Variants were selected for sequencing when the melting profile of at least one of each duplicate sample deviated by more than 10% from control reference samples. Either two or three control reference DNA samples of known wild-type sequence were routinely run in parallel with test samples and second derivative melting data (difference plot) was obtained by analysing samples relative to reference samples. PCR products showing variant melting profiles were sequenced using big dye terminators by standard methods using the same primers used in HRM-PCR. Dye was removed from amplicons prior to sequencing by column clean-up using the QIA PCR purification kit (50) (Qiagen) according to the manufacturers instructions. Sequence traces were screened for mutations using Mutation Surveyor software (Softgenetics PA 16803).

For digital HRM, 240 pg of tumor genomic DNA was aliquoted to a mastermix containing sufficient reagent for PCR amplification of 40 individual samples, each of 20 ul reaction volume. Forty individual reactions, containing on average 6 pg of genomic DNA equivalent to the amount of DNA in a single diploid cell, were analysed by HRM. 60 pg of DNA, from each of two individual control reference DNA samples, was aliquoted to a mastermix sufficient for ten individual PCR reactions thereby generating reactions tubes each comprising 6 pg of reference DNA. Both the 20 reference sample and 40 tumor sample reactions were run simultaneously on the Rotorgene 6000 using the conditons described above appropriate for the exon analysed. The number of PCR cycles was increased from 45 to 50 to account for the lesser amount of DNA in each reaction. Amplicons successfully amplified were subjected to HRM using the conditions described above. The confidence percentage threshold of the Rotorgene 6000 software was set at 90% for variant scoring. Dye removal and sequencing were as described above.

## Results

The SMARCB1 gene encodes nine exons. Primer sequences designed to flanking intronic sequences were used to amplify tumor DNA in the presence of fluorescent dye and the amplicon was then subjected to HRM within the same tube. PCR and high resolution melting conditions were initially explored and optimized with several commercially available fluorescent dyes and HRM reagent mixes, including Syto 9 (Invitrogen), LC Green plus (Idaho Technologies) and EvaGreen dye supplied with the HRM Sensimix (Quantace). We were unable to amplify GC-rich exon 1 in the presence of the dyes Syto 9 and LC Green Plus using the Hot Star Taq polymerase PCR reagent mixes (Qiagen) and obtained inconsistent melting curves for exon 4, however all other exons gave consistent melting profiles with these dyes. Using LC Green Plus the reaction annealing temperatures were increased by 2°C relative to those used with the other dyes and PCR optimization required the addition of MgCl_2 _for exons 2, 3, 4, 5, 6, 7 and 9 to a final concentration of 2 mM and for analysis of exon 8, MgCl_2 _was added to a final concentration of 2.5 mM. 1 × Q-Solution (Qiagen) was added for amplification of exons 2, 8, and 9, and 0.5× Q solution was added for amplification of exons 3 and 4. However using HRM Sensimix with the EvaGreen dye we were able to readily amplify all SMARCB1 exons without the addition of denaturing reagents or MgC1_2 _(as described in methods above) and subsequently used this reagent for all mutation screening and method validation.

For method validation, all SMARCB1 exons in fourteen samples (140 amplicons in total) were screened by both HRM and direct sequencing. These fourteen samples comprised DNA isolated from twelve possible rhabdoid tumors and two blood samples. Melting profiles were compared to those obtained for three unrelated control DNA samples representing DNA isolated from peripheral blood, and were scored as wild-type or variant. Reactions were set up in duplicate and an amplicon was scored as variant if the confidence percentage threshold of the Rotorgene 6000 software yielded a score of <90% for at least one of duplicate technical replicates, where the melting curves for each test sample amplicon were normalized consecutively to each of the selected control samples.

**Figure 1:**
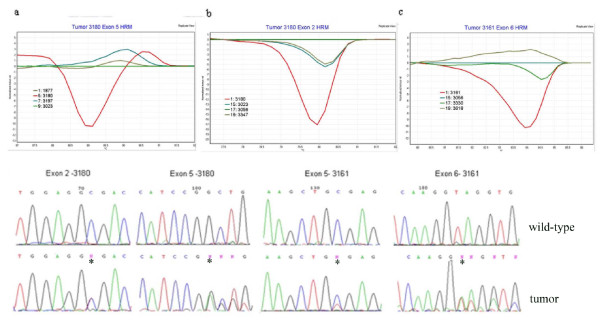
**SMARCB1 mutation screening by HRM in tumors 3180 and 3161**. Figure 1 shows HRM difference plots for; (a) exon 5 in tumor 3180, (b) exon 2 in tumor 3180 and (c) exon 6 in tumor 3161. The melting curves associated with the identified sequence variations (shown in red) were clearly distinguishable from the control DNA samples (green lines). HRM data for exon 5 in tumor 3161 is shown in Figure 2a. The melting curves shown represent the average melting data of grouped duplicate samples. (d) sequence traces for each mutation detected. The mutation position is indicated by *.

A summary of the results obtained for HRM and direct amplicon sequencing is shown in Table 2. Two tumors, 3161 and 3180, carried two distinct mutations. In one tumor, (3161), mutations were identified in both exon 5 and exon 6 and in another tumor, (3180), mutations were identified in both exon 2 and exon 5. These tumors did not have allele loss for chromosome 22q suggesting that each SMARCB1 allele was independently affected by a mutation. In tumors 3331 and 3641, single mutations were identified. Two of the twelve tumors in the panel, had wild-type amplicon melting profiles, and were subsequently shown to have homozygous deletion of SMARCB1 confirmed on southern blotting. Amplicons analysed from these specimens contained wild-type sequence and were presumably derived from contaminating normal tissue. Figure 1 shows representative mutation screening and sequence data for tumors 3180 and 3161.

**Table 2:** Validation data for SMARCB1 screening by HRM

Exon Analysed	Total Sample Number	Coding sequence mutation identified by direct sequencing	Polymorphism identified by direct sequencing	Wild-type melt profiles	Variant melt profiles	False Positive Melt	False Negative melt
**Exon 1-1**	14	0	0	13	1	1(7.1%)	0

**Exon1-2**	14	0	14 dinuc GC repeat	0	14	0	0

**Exon 2**	14	c157C>T (3180) c157C>T (3641)	0	12	2	0	0

**Exon 3**	14	0	0	13	1	1(7.1%)	0

**Exon 4**	14	0	0	13	1	1(7.1%)	0

**Exon 5**	14	c601C>T (3161) c569-570 ins18 (3180)	0	12	2	0	0

**Exon 6**	14	c795+2delinsATGA (3161)	0	12	1	1 (7.1%)	0

**Exon 7**	14	c933_934insAT (3331)	4 (SNPrs35817983)	10	4	0	0

**Exon 8**	14	0	0	10	4	4 (28.6%)	0

**Exon 9**	14	0	5 (SNP rs5030613)	9	6	1 (7.1%)	0

Validation was performed on the sample series; 3022, 3073, 3074, 3161, 3180, 3327, 3331, 3356, 3441, 3473, 3474, 3567, 3640 and 3641.

The false negative rate for all exons in all fourteen DNA samples was zero, ie samples with normal melt profiles were found to have no sequence variants on sequencing. A dinucleotide GC repeat polymorphism in intron 1, amplified with the Exon 1-2 primer pair, was detected by HRM as variant in all fourteen samples screened. This highly variant polymorphism was detected as variant relative to most selected control samples in which it was also found to be present in variable repeat copy number. All mutations previously detected by direct sequencing were clearly identifiable as shifted melting profiles on HRM. A polymorphism 3' to exon 7, g38509_38510 indel AA (NC_000022) (SNP rs35817983) was detected by HRM in four samples and another polymorphism 5' to exon 9, g47138G>A (NC_000022) (SNP rs5030613) was detected in five samples.

False positive sequence variations were detected in six exons, exon 1-1, exon 3, exon 4, exon 6, exon 8 and exon 9 however the overall false positive rate was low. False positives were not associated with any detectable sequence variation. Three false positives were identified in a single tumor DNA sample (3161) suggesting that DNA quality may have contributed to the observed variation. On review, whereas the Abs 260/280 nm was acceptable for this DNA (1.90), the A260/230 ratio was poor (0.44) suggesting the presence of small molecule contaminants in the DNA preparation.

Mutations identified in all rhabdoid tumors screened including the results of immunohistochemical staining for SMARCB1 protein, are shown in Table 3. This table includes tumors screened by direct sequencing only and tumors screened using both HRM and sequencing. Germline mutation screening was performed on blood from seven cases. However we did not identify any cases with a germ-line mutation in SMARCB1.

**Table 3:** Summary of pathogenic mutations identified in rhabdoid tumors

Tumor	Exon	Mutation relative to ATG codon	Prediction	SMARCB Protein	Site	22qLOH
**1877**	Exon 2	c118C>T	pARG40STOP	Absent	Abdominal	Yes

**1918**		None		Faint	Choroid plexus	Yes

**1938**	Exon 9	c1144delG	Frame shift	Absent	CNS	Yes

**1993**	Exon 3	c325insG	Frame shift	Absent	CNS	ND

**3022**		None		Present	Renal	Yes

**3074**		Deletion of gene	Absent	Absent	CNS	Yes

**3161**	Exon 5	c601C>T	pARG201STOP	Absent	CNS	No
				
	Exon 6	c795+2delinsATGA	Splice donor site disrupted			

**3180**	Exon 2	c157C>T	pARG53STOP	Absent	CNS	No
				
	Exon 5	c569-570ins18	Frame shift			

**3349**		Deletion of gene	Absent	Absent	Liver	No

**3331**	Exon 7	c933_934insAT	Frame shift	Absent	CNS	Yes

**3388**	Exon 9	c1148delC	Frame shift	Absent	CNS	ND

**3641**	Exon 2	c157C>T	pARG53STOP	Absent	CNS	ND

**3803**	Exon 2	c118C>T	pARG40STOP	Absent	CNS	ND

ND; Not determined.

**Figure 2:**
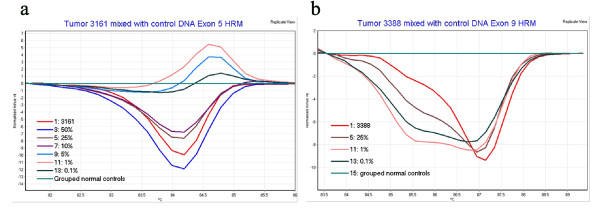
**SMARCB1 mutation detection sensitivity using HRM**. HRM data showing the sensitivity for the detection of specific sequence variants in; (a) exon 5 in tumor 3161, c601C>T (1:10), and (b) exon 9 in tumor 3388, c1148delC (1:1000). The melting curves shown represent the average melting data of grouped duplicate samples. The grouped normal control data was a composite of the duplicate melt data from three distinct biological control DNA samples.

To determine the sensitivity of HRM for detecting specific mutations DNA containing pathogenic sequence variation in SMARCB1 was mixed with control DNA and screened by HRM. Given that many tumors submitted for diagnostic screening may be mixtures of malignant and non-malignant cells it was considered important to establish the reliability of HRM screening for detecting mutations within these settings. DNA samples with identifiable SMARCB1 mutations in exons 2, 3, 5, 6 and 9 were mixed with control DNA to give ratios of mutant to normal DNA of 1:2, 1:4, 1:10, 1:20, 1:100 and 1:1000. These ratios are necessarily approximations because tumor DNA is typically already a mix with DNA from normal tissue associated with tumor biopsies. The true ratio of mutant to normal DNA is difficult to precisely predict and may vary from sample to sample. Nevertheless when tumor DNA was mixed with normal DNA in the proportions described, it was apparent that high detection sensitivities could be achieved (Table 4). The sensitivity for detection was both sequence context and variant dependent. The lowest ratio of mutant to normal DNA for each mutation at which the threshold remained consistently below the 90% confidence threshold in repeat runs, is shown in Table 4. HRM data showing the sensitivity for the detection of specific sequence variants in exon 5 in tumor 3161 (1:10) and in exon 9 in tumor 3388 (1:1000) is shown in Figures 2a and 2b respectively. We were unable to ascertain the detection limit for all mutations due to insufficient availability of tumor DNA, however this analysis demonstrates the sensitivity of HRM for detecting mutations and shows for certain sequence variants a very high sensitivity can be achieved.

**Table 4:** Detection sensitivity for SMARCB1 mutations in mosaic mixes of tumor DNA

Exon	Tumor	Mutation	Proportion of tumor DNA: normal DNA
Exon 2	3180	c157C>T	1:4

Exon 5	3180	c569-570ins18	1:4

Exon 3	1993	c325insG	1:4

Exon 5	3161	c601C>T	1:10

Exon 9	3388	c1148delC	1:1000

Exon 6	3161	c795+2delinsATGA	1:1000

**Figure 3:**
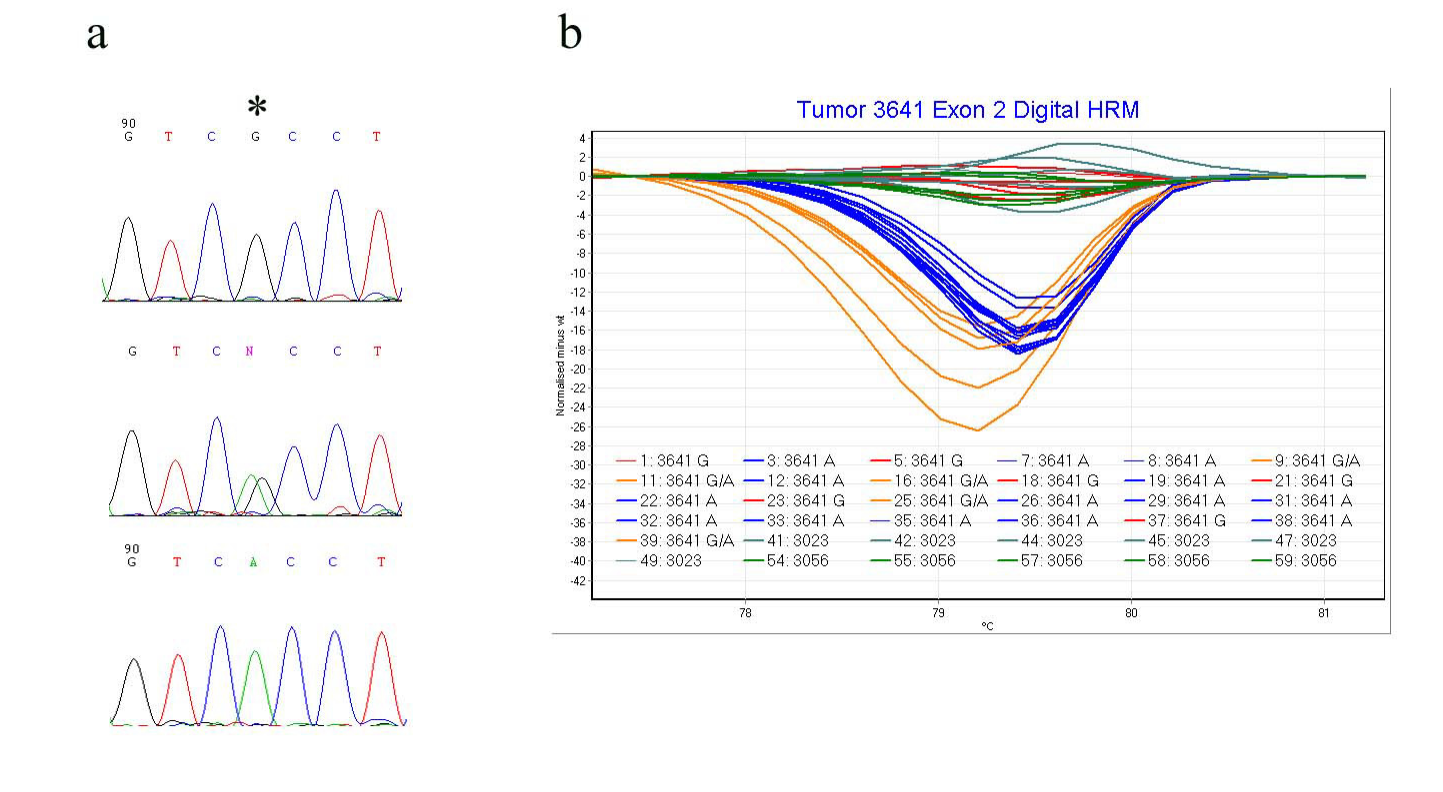
**Isolation of mutations using digital HRM**. Figure 3 shows sequence traces (3a) for genotypes identified by HRM (3b) following dilution of DNA from tumor 3641 and amplification from 6 pg DNA. Each melting curve is derived from a single amplicon. Three genotypes were identified in this tumor as shown in 3b and are depicted by red, blue and orange differential melting curves. Green melting curves represent that of wild-type, non-tumor control DNA. The wild-type genotype in sample 3641 has a G at nucleotide 157 (reverse sequence shown with nucleotide position denoted by *), and the mutant genotype has an A at this position. A sub-population with G/A was also identified.

Although HRM has a high sensitivity for detecting sequence variants, verification of mutations by direct DNA sequencing in the context of tumor and normal tissue DNA mixtures can sometimes be problematic, necessitating cloning and re-sequencing of the cloned amplicon, to confirm the presence of a mutation. We developed a novel strategy combining digital PCR and HRM (digital HRM) to overcome the requirement for cloning. In this method tumor DNA was serially diluted in water such that the final amount of DNA in a single amplification tube was approximately 6 pg, where 6 pg represents the amount of DNA a single diploid cell. Forty individual amplifications from each sample were performed simultaneously alongside ten amplifications from each of two normal controls. Amplicons were then subjected to HRM and the melting profiles of these amplicons were analysed to distinguish wild-type from variant sequences. On average 65% of amplicons were successfully amplified at these low concentrations. Amplicons with wild-type and variant melting curves were sequenced to ascertain their genotype. In the example shown in Figure 3, three genotypes were identified in tumor 3641 with a mutation in exon 2 c157C>T. One genotype depicted by the red melting curves in Figure 3b contained wild-type sequence (G at position 157 in the reverse sequence) and clustered with normal control samples, the second and most predominant genotype (A at position 157 on reverse sequence) clustered together (blue melting curves) and a third less common genotype (G/A) clustered in a third melting group (orange melting curves). The sequence data for each genotype is shown in Figure 3a. We interpret this to show a wild-type genotype derived from normal tissue DNA, an isolated mutant tumor genotype, and a mixed genotype present in a sub-population of amplicons. It is not possible to ascertain the mechanism of tumorigenesis without performing copy number analysis, however given the evidence for 22q deletion in rhabdoid tumor in association with SMARCB1 mutation it is likely that c157C>T is a hemizygous mutation and that the heterozygous genotypes are derived from amplicons where the DNA amplified is derived from both normal and tumor DNA.

## Discussion

In our experience, once validated for a specific gene, HRM is a rapid screening method with high sensitivity. However validation of HRM as a pre-sequencing tool requires extensive optimisation of both reaction conditions and reagents to ensure confidence and uniformity in calling sequence variants. Independent in-house validation by labs contemplating adopting this technology for diagnostic pre-sequencing mutation screening is strongly recommended. Uniformity in DNA extraction protocols is also an important factor in the reliability of HRM.

The false negative rate determined in our series was zero. However the false positive rate was amplicon-dependent and impacted by trace impurities in the DNA. In the diagnostic setting where all variants are sequenced to confirm their true status, false positives do not present a significant problem, however the true false negative rate is more difficult to ascertain unless large tumor series with a diversity of mutations can be examined. In these circumstances other diagnostic parameters including negative SMARCB1 protein staining should be used as a guide to assess the likelihood that pathogenic mutations may have been missed and to the possibility of homozygous deletion of the gene, present in up to 20% of rhabdoid tumors. These deletions will not be detected by either HRM screening nor by direct sequencing and a false negative could be reported in such circumstances. Application of alternative techniques including southern blotting and MLPA is highly recommended for ascertainment of these cases.

## Conclusions

This is the first report describing SMARCB1 mutation screening using HRM. HRM, once established, yields significant savings on analysis time and sequencing costs. While immuno-histochemical staining for SMARCB1 protein has greatly facilitated the diagnosis of rhabdoid tumor, identification of a mutation is necessary for the ascertainment of the germ-line mutational status. This is becoming increasingly relevant in line with reports of associations between SMARCB1 mutation and germ-line predisposition to both rhabdoid tumor and schwannoma, and is an important aspect of clinical management for these patients [12-14]. While we did not identify any rhabdoid tumor cases with germline SMARCB1 mutation in our small series, germ-line predisposition has now been reported in a sufficient number of cases of both rhabdoid tumor and schwannomatosis to warrant routine investigation.

## Abbreviations

(HRM): High resolution melting; (FFPE): formalin-fixed paraffin embedded; (AT/RT): atypical teratoid rhabdoid tumor.

## Competing interests

The authors declare that they have no competing interests.

## Authors' contributions

VD conducted the experiments and analysed the data. CWC performed histological analysis, immunohistochemistry, referred tumor specimens and edited the manuscript. DA participated in project design and coordination. EA designed the project, analysed the data and wrote the manuscript. All authors read and approved the final manuscript.

## Author's information

EA is the head of the Molecular Oncology laboratory, a diagnostic laboratory within the Children's Cancer Centre.

## Pre-publication history

The pre-publication history for this paper can be accessed here:

http://www.biomedcentral.com/1471-2407/9/437/prepub

## References

[B1] Biegel JA Zhou JY Rorke LB Stenstrom C Wainwright LM Fogelgren B Germ-line and acquired mutations of INI1 in atypical teratoid and rhabdoid tumors Cancer research 1999 59 1 74 79 9892189

[B2] Versteege I Sevenet N Lange J Rousseau-Merck MF Ambros P Handgretinger R Aurias A Delattre O Truncating mutations of hSNF5/INI1 in aggressive paediatric cancer Nature 1998 394 6689 203 206 10.1038/28212 9671307

[B3] Judkins AR Burger PC Hamilton RL Kleinschmidt-DeMasters B Perry A Pomeroy SL Rosenblum MK Yachnis AT Zhou H Rorke LB INI1 protein expression distinguishes atypical teratoid/rhabdoid tumor from choroid plexus carcinoma Journal of neuropathology and experimental neurology 2005 64 5 391 397 15892296 10.1093/jnen/64.5.391

[B4] Xiangru Wu VD Algar E Muscat A Bandopadhayay P Ashley D Chow CW Rhabdoid tumour:a malignancy of early childhood with variable primary site, histology and clinical behaviour Pathology 2008 40 664 670 10.1080/00313020802436451 18985520

[B5] Ammerlaan AC Ararou A Houben MP Baas F Tijssen CC Teepen JL Wesseling P Hulsebos TJ Long-term survival and transmission of INI1-mutation via nonpenetrant males in a family with rhabdoid tumour predisposition syndrome British journal of cancer 2008 98 2 474 479 10.1038/sj.bjc.6604156 18087273 PMC2361463

[B6] Biegel JA Fogelgren B Wainwright LM Zhou JY Bevan H Rorke LB Germline INI1 mutation in a patient with a central nervous system atypical teratoid tumor and renal rhabdoid tumor Genes, chromosomes & cancer 2000 28 1 31 37 10.1002/(SICI)1098-2264(200005)28:1<31::AID-GCC4>3.0.CO;2-Y 10738300

[B7] Hulsebos TJ Plomp AS Wolterman RA Robanus-Maandag EC Baas F Wesseling P Germline mutation of INI1/SMARCB1 in familial schwannomatosis American journal of human genetics 2007 80 4 805 810 10.1086/513207 17357086 PMC1852715

[B8] Modena P Lualdi E Facchinetti F Galli L Teixeira MR Pilotti S Sozzi G SMARCB1/INI1 tumor suppressor gene is frequently inactivated in epithelioid sarcomas Cancer research 2005 65 10 4012 4019 10.1158/0008-5472.CAN-04-3050 15899790

[B9] Sevenet N Sheridan E Amram D Schneider P Handgretinger R Delattre O Constitutional mutations of the hSNF5/INI1 gene predispose to a variety of cancers American journal of human genetics 1999 65 5 1342 1348 10.1086/302639 10521299 PMC1288286

[B10] Taylor MD Gokgoz N Andrulis IL Mainprize TG Drake JM Rutka JT Familial posterior fossa brain tumors of infancy secondary to germline mutation of the hSNF5 gene American journal of human genetics 2000 66 4 1403 1406 10.1086/302833 10739763 PMC1288204

[B11] Wittwer CT Reed GH Gundry CN Vandersteen JG Pryor RJ High-resolution genotyping by amplicon melting analysis using LCGreen Clinical chemistry 2003 49 6 Pt 1 853 860 10.1373/49.6.853 12765979

[B12] Fujisawa H Takabatake Y Fukusato T Tachibana O Tsuchiya Y Yamashita J Molecular analysis of the rhabdoid predisposition syndrome in a child: a novel germline hSNF5/INI1 mutation and absence of c-myc amplification Journal of neuro-oncology 2003 63 3 257 262 10.1023/A:1024345221792 12892231

[B13] Hadfield KD Newman WG Bowers NL Wallace A Bolger C Colley A McCann E Trump D Prescott T Evans DG Molecular characterisation of SMARCB1 and NF2 in familial and sporadic schwannomatosis Journal of medical genetics 2008 45 6 332 339 10.1136/jmg.2007.056499 18285426

[B14] Janson K Nedzi LA David O Schorin M Walsh JW Bhattacharjee M Pridjian G Tan L Judkins AR Biegel JA Predisposition to atypical teratoid/rhabdoid tumor due to an inherited INI1 mutation Pediatric blood & cancer 2006 47 3 279 284 10.1002/pbc.20622 16261613

